# Conjunctival Flora in Diabetic and Nondiabetic Individuals

**DOI:** 10.4274/tjo.33230

**Published:** 2015-10-05

**Authors:** Mehmet Adam, Mehmet Balcı, Hasan Ali Bayhan, Ahmet Çağkan İnkaya, Mehmet Uyar, Canan Gürdal

**Affiliations:** 1 Bozok University Faculty of Medicine, Department of Ophthalmology, Yozgat, Turkey; 2 İzzet Baysal State Hospital, Clinic of Infectious Disease and Microbiology, Bolu, Turkey; 3 Hacettepe University Faculty of Medicine, Department of Infectious Disease and Clinical Microbiology, Ankara, Turkey; 4 Necmettin Erbakan University Faculty of Medicine, Department of Public Health, Konya, Turkey

**Keywords:** Conjunctival flora, diabetes mellitus, gram-negative bacteria

## Abstract

**Objectives:**

To evaluate the conjunctival bacterial flora in diabetic patients and nondiabetic subjects.

**Materials and Methods:**

Fifty-three diabetic patients and 43 nondiabetic healthy individuals were included in the study. A specimen was taken from each participant for the study by rubbing a sterile cotton-tipped swab on the inferior palpebral conjunctiva of the right eye. Samples were incubated in blood agar, chocolate agar, eosin methylene-blue lactose sucrose agar and sabouraud 4% dextrose agar. Isolated microorganisms were identified using routine microbiological methods.

**Results:**

Rates for bacterial isolations were determined as 38.5% in diabetic patients and 34.9% in nondiabetic controls. Staphylococcus aureus was isolated in 30% of cases in the diabetic patient group, while 20% tested positive for Escherichia coli, 10% for coagulase-negative Staphylococcus, 10% for Klebsiella pneumoniae and 30% for multiple bacteria. In the non-diabetic group, 53.3% of patients were positive for Staphylococcus aureus while coagulase-negative Staphylococcus was isolated in 26.7%, Klebsiella pneumoniae in 6.7% and multiple bacteria in 13.3% of patients. Although there was no statistically significant difference in the number of isolated bacteria between the diabetic and nondiabetic groups, gram-negative bacterial colonization was significantly higher in diabetic patients (χ2=0.129, p=0.719 and χ2=5.60, p=0.018, respectively).

**Conclusion:**

Gram-negative bacteria are more common in the conjunctival flora of diabetic patients. This should be considered by clinicians when treating ocular infections in diabetic patients.

## INTRODUCTION

The conjunctiva is a transparent mucous membrane lining the internal surfaces of the eyelids and the orbital globe. The surface is covered with stratified, non-keratinized epithelial cells and functions as a barrier against infection. The conjunctival flora is found on the ocular surface of healthy individuals and under normal conditions comprises noninfectious microorganisms. These microorganisms have an important role in the maintenance of normal conjunctival functions and the prevention of ocular infections.^1^

Conjunctival flora begins forming at birth and continues to increase over the lifespan. Flora may vary depending on environment, age, immunity, ocular surface diseases, systemic diseases, climate, region and general hygienic conditions.^2^ The flora of the ocular surface consists more of gram-positive microorganisms.^3^ This microbial colonization of the conjunctiva contributes to making it more difficult for potentially pathogenic microorganisms to colonize the ocular surface. Despite the flora’s defensive element, it can become pathogenic in situations such as after a surgical procedure or when the immune system is compromised.

Diabetes mellitus is a multi-factorial disease that can affect all ocular structures, especially the retina. Diabetic patients have a higher risk of postoperative endophthalmitis than non-diabetic patients.^4^ The flora of the conjunctiva, eyelid and even the nasal mucosa form the majority of pathogenic microorganisms involved in postoperative endophthalmitis.^5^

The aim of this study was to investigate the aerobic bacterial conjunctival flora in diabetic patients and compare it with that of non-diabetic individuals. Evaluating flora elements, which are important pathogens of ocular infections, may lead to the development of better treatment regimes. 

## MATERIALS AND METHODS

The study included 53 diabetic and 43 non-diabetic patients who presented to the Bozok University Ophthalmology Clinic. All diabetes cases were type II. Study participants signed a written consent form indicating their understanding and voluntary participation in the study after being informed in detail. The study was approved by the local ethics committee and was conducted in accordance with the Helsinki Declaration of Human Rights.

Prior to examination, specimens were obtained using a sterile swab by scraping each participant’s lower right fornix, avoiding contact with the eyelid margins and eyelashes and without the use of local anesthesia. This prevented any possible contamination due to the examination. All participants underwent a thorough ophthalmologic examination. Vision was assessed by Snellen chart, then slit-lamp examination of the anterior and posterior segment was performed. Patients with examination findings or history of any of the following were excluded from the study: dry eye or glaucoma and topical medication use for these reasons; blepharitis or anterior segment infection; nasolacrimal duct obstruction; contact lens use; and systemic or topical antibiotic use within the previous 2 months. Patients presenting to the ophthalmology clinic for refraction who did not have blepharitis or anterior segment infection, did not have a history of contact lens use and had normal blood glucose were included in the control group.

Conjunctival specimens were immediately sent to the microbiology laboratory and seeded on blood agar, eosin methylene blue (EMB) lactose sucrose agar, Sabouraud dextrose agar (SDA) and chocolate agar. Cultures on blood agar, chocolate agar and EMB were incubated at 37 °C for 24 hours; cultures on SDA were incubated at 25 °C for 2 weeks. After incubation, gram staining was performed. Gram-positive cultures were identified by coagulase and catalase testing; gram-negative cultures were identified using the API 20E bioMerieux (bioMerieux Canada, Inc.) identification system. Anaerobic bacteria were not investigated in this study.

Data were analyzed using SPSS version 15.0 statistical analysis package. Descriptive data were expressed as number (n) and percentage (%). Data were analyzed for normal distribution. Categorical data were compared using the chi-square test and Student’s t-test was used in the analysis of continuous variables. Spearman correlation analysis was used to evaluate correlation. Level of significance was accepted as α=0.05.

## RESULTS

The diabetic group included 34 (64.15%) women and 19 (35.85%) men; the control group included 26 (60.45%) women and 17 (39.55%) men. The average age was 53.94±9.24 years (range, 38-70 years) in the diabetic group and 55.23±10.97 years (range, 40-71 years) in the control group. There were no significant differences between the groups in terms of demographic characteristics (gender, p=0.71; age, p=0.89). Among the diabetic group, 44 individuals (83%) were using an oral antidiabetic and 9 (17%) were using insulin; the mean duration of diabetes was 7.81±5.77 years (range, 1-20 years). Twelve patients (22.6%) had diabetic retinopathy (DR).

Bacterial growth occurred in the conjunctival cultures of 38.5% of diabetic patients and 34.9% of non-diabetic individuals. The following bacteria were identified in cultures from the non-diabetic group: Staphylococcus aureus (gram-positive cocci) in 8 cultures (53.3%); coagulase-negative Staphylococci (CNS, gram-positive cocci) in 4 cultures (26.7%); Klebsiella pneumoniae (gram-negative bacilli) in 1 culture (6.7%) and more than one species of bacterium in 2 cultures (13.3%). In the diabetic group, the following bacteria were identified: Staphylococcus aureus in 6 cultures (30%); Escherichia coli (gram-negative bacilli) in 4 cultures (20%); CNS in 2 cultures (10%); Klebsiella pneumoniae in 2 cultures (10%); and more than one species of bacterium in 6 cultures (30%). There was no difference between the diabetic and non-diabetic groups in the ratio of positive cultures (chi-square=0.129, p=0.719), though gram-negative bacteria were more common in diabetics than in non-diabetics (chi-square=5.60, p=0.018, Table 1). The positive culture ratio was 33.3% in proliferative diabetic retinopathy (PDR) patients and 55.6% in patients using insulin; however, neither group showed a significant difference from the control group in the frequency of positive cultures (chi-square=0.99, p=0.609 and chi-square=2.73, p=0.098, respectively). There was no correlation between diabetes duration and positive cultures (r=0.218, p=0.11).

## DISCUSSION

In this study, the conjunctival flora of diabetic patients and healthy individuals were compared. Specimens obtained for this purpose were seeded on different media. The diabetic group showed no significant difference in frequency of bacterial growth compared to the control group, but yielded more gram-negative bacterial cultures.

Although the conjunctival flora forms a defensive barrier against infection, it also includes major pathogens of ocular infections. In healthy individuals, the conjunctival flora is frequently comprised of same microorganisms as the skin flora.^6^ Gram-positive bacteria constitute the main elements of bacterial floral, though the positive culture rate and microorganisms grown show diversity.^2,3^

Higher rates of bacterial colonization are expected in situations that weaken the immune system such as diabetes, advanced age, and corticosteroid use.^7^ However, it has been reported in the literature that infections that substantially suppress the immune system, such as HIV, don’t cause significant changes in positive culture frequency or the variety of bacteria found in conjunctival cultures.^8,9^ Similar changes in diabetic patients have also been reported in the literature. Suto et al.^10^ studied 579 individuals and found a unilateral positive culture rate of 39.2% with CNS as the major bacterial flora element. In the same study, the rate of gram-negative bacteria was 5.9% and the most common gram-negative bacterium was Escherichia coli. In our study, gram-positive bacteria were the major bacterial flora element and among the gram-positive cultures, Staphylococcus aureus was most common. CNS was the second most common microorganism in the non-diabetic group and the third most common in the diabetic group. Similar to our study, Birinci et al.^11^ identified Staphylococcus aureus as the most common bacterial flora element in diabetic patients.

We did not find a significant difference in bacterial isolation rate between the diabetic and non-diabetic groups. Furthermore, there was no difference in positive culture growth frequency between the PDR group and the control group (33.3% and 34.9%, respectively). Despite reports in the literature of differences in bacterial growth frequency between individuals with and without diabetes, other researchers have found no such difference. Karimsab and Razak^12^ found a higher positive culture rate in their diabetic group compared to their non-diabetic group (34% versus 24%, respectively). Higher frequency of positive cultures has also been observed in PDR patients. Arbab et al.^13^ observed a positive culture rate of 75% in their PDR group compared to 20% in patients without retinopathy, and Staphylococcus epidermidis was the most common isolate. In contrast, Suto et al.^10^ found no difference in the frequency of positive cultures between diabetic and non-diabetic patients and no relationship between positive culture frequency and the presence of diabetic retinopathy. These conflicting results may be attributable to the differences in the DR rate between studies. The DR rates in the aforementioned studies were 86.77% for Karimsab and Razak,^12^ 74.8% for Arbab et al.^13^ and 8.29% for Suto et al.^10^ In our study, the rate of DR was 22.6%.

Another factor that may affect the conjunctival flora is type of hypoglycemic therapy. In our study, positive culture rates were higher among patients using insulin compared to the control group, though the difference was not statistically significant. Arbab et al.^13^ observed no relationship between hypoglycemic therapy and bacterial growth frequency and also found that the duration of diabetes had no effect on positive culture rates. Similarly, Martins et al.^14^ grouped patients by diabetes duration (more or less than 5 years) and found that the duration of diabetes had no effect on the frequency of positive cultures or the variety of flora bacteria. Martins et al.^14^ also found that hypoglycemic therapy, age and gender had no effect on culture results.

In this study we aimed to compare the conjunctival flora of diabetic patients and healthy individuals. The most important result of our study is the higher frequency of gram-negative bacterial isolates in the diabetic group. Rubio et al.^15^ evaluated the conjunctival flora of patients prior to cataract surgery and found that diabetic patients had a higher prevalence of Klebsiella pneumoniae and gram-negative diplococci than non-diabetic patients. Philips and Tasman16 found that gram-negative bacteria account for the higher prevalence of endophthalmitis in diabetics compared to non-diabetics and that gram-negative microorganisms result in a poorer endophthalmitis prognosis. In a study of endogenic endophthalmitis by Lim et al.^17^ including 53 patients, gram-negative bacteria were detected in 54.38% of cases. Klebsiella pneumoniae was the most common gram-negative bacterial isolate (45.61%) and diabetes was determined to be the most significant underlying risk factor. Similarly, gram-negative agents are noteworthy in other infections in diabetics. Zhang et al.^18^ showed that the prevalence of gram-negative bacteria was four times higher in diabetics with chronic rhinosinusitis than in a control group. In another study of patients with diabetic foot ulcers, gram-negative bacteria were isolated from 65.1% of positive cultures.^19^

Gram-positive and gram-negative bacteria differ in their sensitivity to antibiotics. Although antibiogram was not performed in this study, there are many such studies in the literature. Coşkun et al.^3^ found that among conjunctival isolates of Staphylococcus aureus, 91.1% were sensitive to ofloxacin and 86.6% to ciprofloxacin, while only 8.8% were sensitive to penicillin G; 28.8% of the isolates were methicillin-resistant Staphylococcus aureus and among these cultures, 38.5% showed sensitivity to ofloxacin or ciprofloxacin. In the same study, sensitivity of isolated Staphylococcus epidermidis cultures to ofloxacin and ciprofloxacin was 92.5% and 91.5%, respectively. Suto et al.^10^ found a higher prevalence of methicillin-resistant CNS in diabetic patients and reported that resistance rates of 14% to levofloxacin and 17.9% to tobramycin in the isolates they obtained. Gupta et al.^20^ found that in all gram-positive cultures isolated from endophthalmitis cases were sensitive to vancomycin and all gram-negative cultures were sensitive to ceftazidime. Long et al.^21^ investigated endophthalmitis following trauma and found the prevalence of gram-negative to be 29.1%, with Pseudomonas aeruginosa and Escherichia coli as the most common isolates. Furthermore, due to the increasing frequency of multiple antibiotic resistance in gram-negative bacteria, they recommended using ciprofloxacin, tobramycin and cephalosporin together in cases of Pseudomonas aeruginosa-related endophthalmitis.

## CONCLUSION

Gram-negative bacteria Escherichia coli and Klebsiella pneumoniae were detected in higher ratios in the conjunctival flora of diabetic patients. Considering that flora elements may be important pathogens in ocular infections, treatment approaches to gram-negative bacteria should not be ignored in cases of ocular infections in diabetics.

## Figures and Tables

**Table 1:**
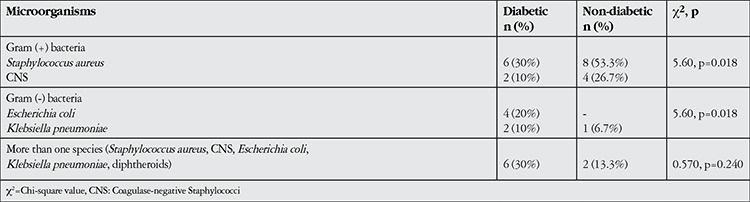
Microorganisms isolated from the conjunctivae of diabetic and non-diabetic patients
